# An Internet-Based Multi-Approach Intervention Targeting University Students Suffering from Psychological Problems: MindBlooming

**DOI:** 10.1192/j.eurpsy.2023.147

**Published:** 2023-07-19

**Authors:** Raffaella Calati, Ionut Daniel Fagadau, Davide Ginelli, Fabio Madeddu, Jorge Lopez-Castroman, Daniele Romano, Alessandro Gabbiadini, Emanuele Preti, Daniela Micucci

**Affiliations:** ^1^Department of Psychology, University of Milan-Bicocca, Milan, Italy; ^2^Department of Adult Psychiatry, Nîmes University Hospital, Nîmes, France; ^3^Department of Informatics, Systems and Communication (DISCo), University of Milano-Bicocca, Milan, Italy; ^4^IGF, CNRS-INSERM, Université Montpellier, Montpellier, France; ^5^CIBERSAM, Madrid, Spain

## Abstract

**Abstract:**

The young adult can experience psychological difficulties related to the delicate phase of life he/she is going through. Many of them show psychological difficulties but only a small portion receive the needed care. In this regard, internet-based interventions represent an important resource.

MindBlooming is a seven-week intervention delivered through a mobile application for university students with mild to moderate psychological difficulties. The application is the result of the interdisciplinary work between the Department of Psychology and the Department of Informatics, Systems and Communication of the University of Milan-Bicocca.

The intervention focused on symptoms of depression, anxiety, sleep problems, self-destructive thoughts, job- and study-related stress and burnout, and chronic pain. It is based on different approaches (multi-approach), primarily psychoeducation, Cognitive-Behavioral Treatment (CBT), and third-wave CBT. During the first pilot study, the intervention consisted of a 7-week treatment on two problematic areas according to each students’ personal needs, identified through an initial assessment. The achieved results are promising especially in terms of interest shown by students. However, we encountered technical problems that hampered the pilot study. A second pilot will be performed to further test the application.

The MindBlooming project will be discussed in light of the need for further multidisciplinary research that confirms how biomarkers can be sensitive to non-pharmacological internet-based interventions.

**Keywords:**

cognitive-behavioral treatment; internet-based intervention; university students.

**Disclosure of Interest:**

None Declared

